# Prevalence of Gingival Biotypes among Young Dentate North Indian Population: A Biometric Approach

**DOI:** 10.5005/jp-journals-10005-1343

**Published:** 2016-06-15

**Authors:** Manu Rathee, Polsani L Rao, Mohaneesh Bhoria

**Affiliations:** 1 Senior Professor and Head, Department of Prosthodontics and Crown and Bridge, Pandit Bhagwat Dayal Sharma Post Graduate Institute of Dental Sciences, Rohtak, Haryana, India; 2 Professor and Head, Department of Prosthodontics and Crown and Bridge, Army College of Dental Sciences, Secunderabad, Telangana, India; 3 Demonstrator, Department of Prosthodontics and Crown and Bridge, Pandit Bhagwat Dayal Sharma Post Graduate Institute of Dental Sciences, Rohtak, Haryana, India

**Keywords:** Gingival biotype, Gingival sulcus, Gingival thickness, Periodontal probe.

## Abstract

**Aim:** To evaluate the prevalence of various gingival biotypes and to corroborate gingival thickness and gingival biotypes across tooth type, site, and gender.

**Materials and methods:** A cross-sectional study was conducted across systemically healthy subjects. A systematic clinical evaluation for gingival biotypes and gingival thicknesses was recorded by modified Iwanson’s gauge, to the nearest 0.1 mm, probing the gingival sulcus at the midfacial aspect of maxillary and mandibular central incisors and first molars. All measurements were made across a total of 920 sites in 115 subjects (69 female and 46 male) based on gingival transparency and were statistically analyzed.

**Results:** A significant agreement on the reproducibility of the measurements was noted. The median overall gingival thickness was recorded at 0.75 mm with interquantile difference of 0.39 mm. The thin biotype variant showed across the ranges of 0.3 to 0.6 mm of gingival thicknesses and thick biotype variant across the ranges of 1.0 to 1.2 mm, with more prevalence in anterior and posterior site respectively. Moreover, for gingi-val thickness of 0.7 mm, the probe visibility showed tendency toward both thin/thick biotype variant in both anterior and posterior segments. The disposition of male participants toward thick biotype and female participants toward the thin biotype variant has been noted.

**Conclusion:** Within the limitations of the current study, our data support the traditional hypothesis of two main gingival biotypes as distinguishable by gingival transparency. In addition, we provide evidence of existence of intermediate biotypes with respect to gingival thickness. These findings can be utilized as objective guidelines for determination of biotype and can be implicated in many dental operative procedures.

**How to cite this article:** Rathee M, Rao PL, Bhoria M. Prevalence of Gingival Biotypes among Young Dentate North Indian Population: A Biometric Approach. Int J Clin Pediatr Dent 2016;9(2):104-108.

## INTRODUCTION

Understanding gingival aspect of restorative dentistry is important in harmonizing esthetics and biological function. Several studies have been conducted to identify different combinations of morphometric data related to soft and hard tissue architecture existing in natural dentition. In these reports, “gingival biotypes” have been identified and described as the thickness of gingiva in the faciopalatal dimension. All these studies proposed the existence of two types of gingival biotype, namely thin and thick.^1^

The identification of the gingival morphology is considered important because differences in soft and hard tissue architecture have shown to exhibit a significant impact on the final esthetic outcome of restorative therapy, periodontal therapy, root coverage procedures, and implant esthetics.^2^ Various methods have been proposed to measure gingival thickness, such as direct measurements, probe transparency (TRAN), ultrasonic devices, and cone-beam computed tomography (CBCT).^3-5^ The use of simple and reliable methods to identify the gingival biotype in clinical practice would be advantageous as this would help to tune the treatment for the individual and predict its specific outcome. Probe visibility through gingival sulcus has been strongly associated with clinical classification of thin biotype, while inability to visualize has been associated with clinical classification of thick biotype.^6^

Although identified in Caucasian population,^7^ gingival biotype applicability to Indian population cannot be corroborating as difference is associated racially and genetically. There is a paucity of evidence correlating the accuracy of these techniques used to ascertain gingival thickness and gingival biotype. The purpose of this article is to present a reproducible, simple method to discriminate gingival biotype based on the gingival transparency with simultaneous measurement of the gingival thickness. Also, there has not yet been an objective classification of gingival tissue based on thickness to identify different gingival biotypes. Hence, the purpose of the present study was to identify the prevalence of gingival biotypes and categorize gingival biotype based on measured gingival thicknesses across tooth type, site, and gender.

## MATERIALS AND METHODS

A cross-sectional study was conducted across a total of 115 (69 females and 46 males) systemically healthy individuals with maintainable oral hygiene. The subjects with history of periodontal flap surgery and orthodontic treatment were excluded. Other exclusion criteria included were prosthetic crowns, abrasion, erosion, caries, or restorations involving the cervical margin of maxillary and mandibular central incisors and first molars. The study protocol was approved by the Institutional Ethical Committee. The study was ethically conducted in accordance with the declaration of Helsinki. All the subjects were fully informed of the investigation and signed informed consent form in Hindi/English, as convenient, prior to examination, was obtained. A total of 920 sites were evaluated by probing the gingival sulcus at the midfacial aspect of maxillary and mandibular central incisors and first molars.

**Fig. 1:**
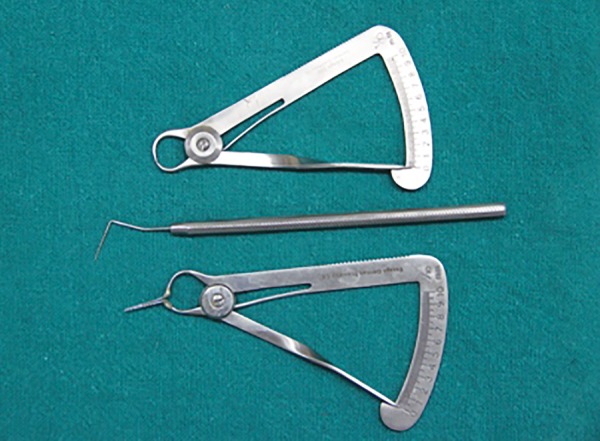
Modified Iwanson’s gauge (Iwanson’s gauge with calibrated periodontal probe tip)

**Fig. 2:**
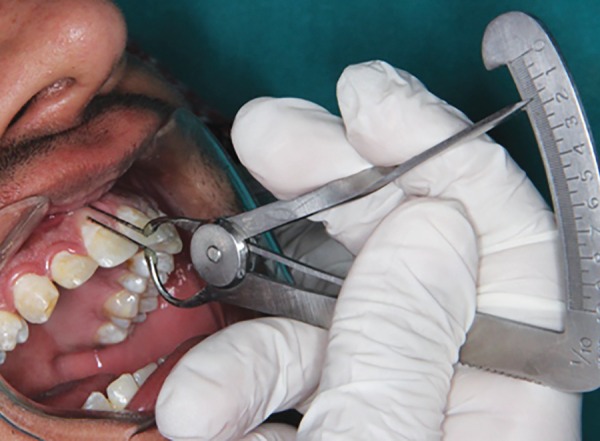
Gingival biotype identified using modified gauge tip

**Fig. 3:**
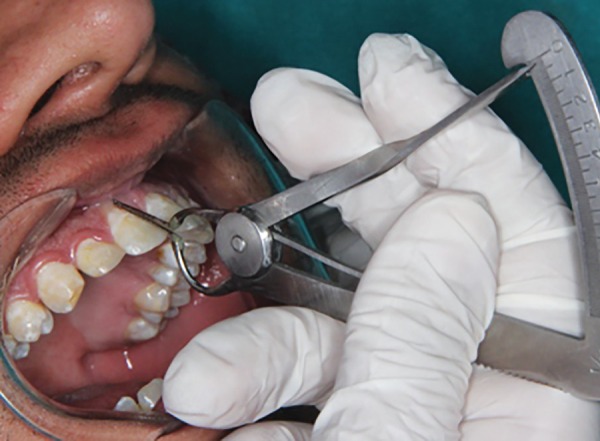
Direct measurement of gingival thickness using modified gauge

Measurement was made with an innovative measurement gauge which was a modified Iwanson’s wax gauge (Essago, Sai Praneet Impex Pvt. Ltd, Navi Mumbai) with attached William Periodontal Probe Calibrated tips (Fig. 1). During the measurement, the transparency of the tip through gingival tissue and thickness of gingival tissue, nearest to 0.1 mm, were made conveniently using single instrument (Figs 2 and 3). Two clinical parameters were systematically evaluated and recorded by one investigator.

*Gingival biotype:* Measured based on the transparency of outline of the underlying gauge tip through gingival tissue. If visible, it was categorized as thin; if not, it was categorized as thick.

*Gingival thickness:* This was calculated at the midfacial region of respective teeth simultaneously with probing while maintaining the direction and position of probe tip.

The data under analysis were organized broadly into two groups, i.e., visible and nonvisible (based on probe tip visibility through gingival sulcus), across tooth type, site, and gender. The intraexaminer repeatability was performed with all clinical examinations. Every first volunteer out of 10 was reexamined after the first recording by the same clinician. For all variables, intraexaminer repeatability was evaluated using Pearson’s correlation coefficient. The median and interquartile differences were used as measure of central tendency and variance respectively. Significant disparities across genders were assessed using the Mann-Whitney test at significance level of p < 0.05. Fisher’s exact test was adopted to evaluate the impact of gender on gingival thickness.

## RESULTS

**Graph 1:**
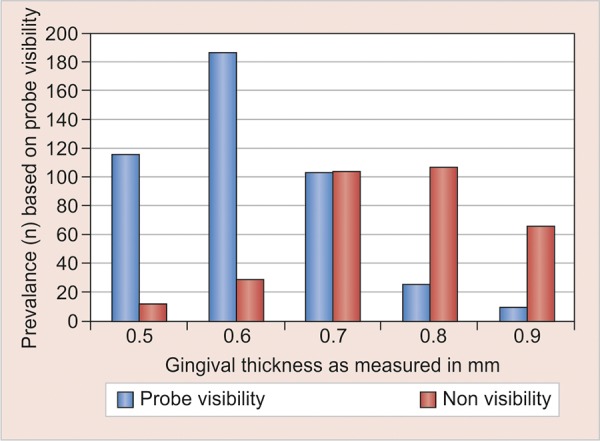
Frequency distribution of different gingival thickness based on visibility of periodontal probe through gingival sulcus

The reproducibility of the measurements was evaluated in 10 volunteers. A strong positive Pearson’s correlation coefficient of 0.8478 (p < 0.0001) was noted. The method to evaluate the gingival thickness and biotype proved to be highly reproducible. The frequency distribution of the gingival thickness showed the biotype was thin (100%) when the gingival thickness was 0.3 and 0.4 mm and thick (100%) when the gingival thickness ranges between 1.0 and 1.2 mm with more prevalence in anterior and posterior sites respectively. For gingival thickness of 0.5 and 0.6 mm, more prevalence occurs in anterior segment and the probe visibility showed tendency toward thin biotype variant. For gingival thickness of 0.8 and 0.9 mm, more prevalence occurs in posterior segment, and the probe visibility showed tendency toward thick biotype variant. Moreover, for gingival thickness of 0.7 mm, the probe visibility showed tendency toward both thin/thick biotype variant in both anterior and posterior segments (Graph 1) (Tables 1 and 2).

The median overall gingival thickness was recorded at 0.75 mm with interquantile difference of 0.39 mm. Overall, 70 to 73% of central incisor showed tendency toward thin biotype variant compared with only 23 to 28% with thick biotype variant. Moreover, 70 to 73% of mandibular first molar showed disposition toward thick biotype variant compared with only 27 to 30% of thin biotype variant. Maxillary first molar showed the most variability in probe visibility with respect to biotype (Tables 3 and 4).

**Table 1:** **Table 1:** Frequency distribution of gingival biotype measurement

		*Transparency of probe tip (%)*							
*Gingival thickness measured in mm*		*Visible*				*Nonvisible*			
		*n*		*%*		*n*		*%*	
0.3-0.4		54		5.86		0		0	
0.5-0.6		303		32.93		38		4.13	
0.7		103		11.19		104		11.3	
0.8-0.9		34		3.69		188		20.43	
1.0-1.2		0		0		96		10.43	

**Table 2:** **Table 2:** Frequency distribution of gingival biotype measurement across tooth type

		*Transparency of probe tip (%)*															
*Gingival* *thickness* *measured* *in mm*		*Visible (thin biotype)*								*Nonvisible (thick biotype)*							
		*Central* *incisor*				*First molar*				*Central* *incisor*				*First molar*			
		*n*		*%*		*n*		*%*		*n*		*%*		*n*		*%*	
0.3-0.4		47		10.21		7		1.52		0		0		0		0	
0.5-0.6		236		51.3		67		14.56		30		6.52		8		1.73	
0.7		37		8.04		66		14.34		43		9.34		61		13.26	
0.8-0.9		9		1.95		25		5.43		47		10.21		141		30.65	
1.0-1.2		0		0		0		0		15		3.26		81		17.6	

The data on gingival thickness were significantly different between male and female participants based on probe visibility and nonvisibility (p = 0.02 and p = 0.002 respectively). Overall, the probe visibility and nonvisibility across gender showed more prevalence of male participants toward thick biotype variant, whereas female participants showed more disposition toward the thin biotype variant (Table 5). Fisher’s exact statistics showed highly significant impact of gender on gingival thickness (p = 0.0002).

**Table 3:** **Table 3:** Probe visibility based on tooth type across anterior site

*Tooth type*		*Probe visibility*			
*Anterior region (n = 115)*		*Yes*		*No*	
*Maxillary right central incisor*					
No. of teeth		82		33	
% visibility		71.30%		28.69%	
*Maxillary left central incisor*					
No. of teeth		81		34	
% visibility		70.43%		29.56%	
*Mandibular right central incisor*					
No. of teeth		83		32	
% visibility		72.17%		27.82	
*Mandibular left central incisor*					
No. of teeth		88		27	
% visibility		76.52%		23.47%	

**Table 4:** **Table 4:** Probe visibility based on tooth type across posterior site

*Tooth type*		*Probe visibility*			
*Posterior region (n = 115)*		*Yes*		*No*	
*Maxillary right first molar*					
No. of teeth		52		63	
% visibility		45.21%		54.78%	
*Maxillary left first molar*					
No. of teeth		51		64	
% visibility		44.34%		55.65%	
*Mandibular right first molar*					
No. of teeth		31		84	
% visibility		26.95%		73.04%	
*Mandibular left first molar*					
No. of teeth		34		81	
% visibility		29.56%		70.43%	

**Table 5:** **Table 5:** Frequency distribution for gingival thickness across gender

		*Male participants* *(n = 46) total site* *(n = 368)*				*Female participants* *(n = 69) total site* *(n = 552)*			
*Transparency of* *periodontal probe*		*n*		*%*		*n*		*%*	
Visible/thin		152		41.3		330		59.78	
Nonvisible/thick		216		58.69		222		40.21	

## DISCUSSION

Achievement of optimal esthetic can be difficult due to inherent different topography of surrounding hard and soft tissue of the natural dentition under individual clinical scenario. An important consideration must be given to the soft tissue which is usually modified to mimic the lost natural emergence profile and contour in individual clinical need to achieve a successful esthetic outcome.^8^ In this regard, the gingival biotypes have been identified and stated to be thick or thin.

The use of simple and reliable methods to identify the gingival biotype in clinical practice would be advantageous as this could help to tune the treatment for the individual and predict its specific outcome. Patients with a thin biotype seem at risk for esthetic failure and therefore need to be accurately identified. Unlike thin gingival biotype, the thick gingival biotype is an important factor for a successful esthetic treatment outcome.^9^ In this regard, an accurate identification of these high-risk patients is warranted. Usually, simple visual inspection was used in clinical practice and even in research to lift out these high-risk patients. However, the precision of this method has never been documented.^4^ Various methods were proposed to measure gingival tissue thickness. These include direct measurements, TRAN, ultrasonic devices, and CBCT. In the direct method,^4^ the tissue thickness was measured using a periodontal probe. When the thickness was 1.5 mm, it was categorized as a thick biotype. When the thickness was <1.5 mm, it was considered a thin tissue biotype. However, this method of measurement had several inherent limitations, such as the precision of the probe, which is to the nearest 0.5 mm, the angulation of the probe during the transgingival probing, and the distortion of the tissue during probing. In the TRAN technique, the gingival biotype was considered thin when the outline of the periodontal probe showed through the gingival margin from inside the sulcus. The biotype was considered thick if the probe did not show through the gingival margin. Also, a noninvasive ultrasonic device was used to measure gingival thickness. Although this method proved to be reproducible, it had several limitations. Most importantly, drawbacks include difficulties in maintaining the directionality of the transducer, unavailability of the device, and high costs. Cone-beam computed tomography scans were used to visualize and measure the thickness of both hard and soft tissues.^5^ However, to the best of our knowledge, there is a paucity of evidence comparing the accuracy of these techniques used to ascertain tissue thickness.

Hence, the present cross-sectional study was conducted to evaluate the prevalence of varying gingival biotypes and to corroborate gingival thickness and gin-gival biotypes across tooth type, site, and gender using a combination of two techniques, i.e., TRAN technique and direct measurement. A systematic clinical evaluation for gingival biotypes and gingival thicknesses was recorded by modified Iwanson’s gauge and a significant agreement on the reproducibility of the measurements by this technique was noted. The method to evaluate the gingival thickness and biotype proved to be highly reproducible. Advantages of using modified measurement gauge were evaluation, categorization of biotype, and measurement of thickness of gingiva done at same time and measurement made nearest to 0.1 mm to confirm accuracy. Also, two clinical parameters could be systematically evaluated and recorded by one clinician. Overall, categorizing gingival biotype would be convenient and easier. The inclusion of maxillary and mandibular central incisors and first molars as reference teeth was done because differences between biotypes are most explicit for these teeth and because their specific features are easily found in other parts of the dentition.^10,11^ Midfacial gingival sulcus was chosen because it is unlikely to be obstructed by the facial bone level. Furthermore, it is comparable to the locations used during assessment by periodontal probe.^12^

Within the limitation of the current investigation, our data support the traditional hypothesis of two main gingival biotypes as distinguishable by gingival transparency. Previous studies have already shown considerable variation between individuals with regard to the morphological characteristics of the periodontium. Already the existence of distinct morphotypes of so-called periodontal biotypes was suggested. Later on, the specific features of these biotypes were well defined.^13,14^ In addition, we provide evidence of the existence of intermediate biotypes with respect to gingival thickness, as for gingival thickness of 0.7 mm, the probe visibility showed tendency toward both thin/thick biotype variant in both anterior and posterior segments.

This present study showed median overall gingival thickness was recorded at 0.75 mm with interquan-tile difference of 0.39 mm. The thin biotype variant showed across the ranges 0.3 to 0.6 mm of gingival thicknesses and thick biotype variant across the ranges of 1.0-1.2 mm, with more prevalence in anterior and posterior sites respectively. However, previous studies have defined the thin tissue biotype as a gingival thickness of <1.5 mm, and the thick tissue biotype was referred to as having a tissue thickness >2 mm.^4^ This cross-sectional study assessed the gingival transparency by assessing the visibility of periodontal probe tip through gingival sulcus, as evaluated in the Caucasian population. However, in the Asian population, the presence of pigmented gingiva^15^ sometimes hampers the correct identification of gingival biotype based on TRAN. This study corroborated possible relationship between gingival biotype and gingival thickness. Hence, measuring gingival thickness at free gingival margin could be a reliable predictor for determining the gingival biotype in pigmented gingiva. The data across gender showed gingival thickness was significantly different between male and female participants based on probe visibility and nonvisibility, respectively. Overall, the probe visibility and nonvisibility across gender showed more prevalence of male participants toward thick bio-type variant, whereas female participants showed more dispositions toward the thin biotype variant. Previous studies favored that the male participants had thicker gingiva to conceal the periodontal probe when compared with females.

Finally, although this study supports the traditional hypothesis that two main different gingival biotypes exist, the inclusion of the intermediate biotype variant is recommended, as at particular gingival thickness of 0.7 mm gingival transparency showed prevalence of both thin and thick biotype.

## CONCLUSION

Within the limitations of the present study, the following three conclusions were drawn:

1. A combined method used to evaluate the gingival thickness and biotype proved to be highly reproducible.
2. The current investigation corroborates existence of three main gingival biotypes as distinguishable by gingival transparency, that is, thin, intermediate, and thick biotype variants.
3. An objective biometric-based categorization of gingival biotype based on gingival thicknesses and transparency has been presented.
